# Is It Still Time for Safety Walkaround? Pilot Project Proposing a New Model and a Review of the Methodology

**DOI:** 10.3390/medicina60060903

**Published:** 2024-05-29

**Authors:** Michela Ferrara, Natascha Pascale, Mauro Ciavarella, Giuseppe Bertozzi, Angela Pia Bellettieri, Aldo Di Fazio

**Affiliations:** 1 Department of Anatomical, Histological, Forensic and Orthopaedic Sciences, Sapienza University of Rome, Viale Regina Elena 336, 00185 Rome, Italy; michelaferrara13@gmail.com (M.F.); dr.mauro.ciavarella@gmail.com (M.C.); 2 SIC Medicina Legale, Via Potito Petrone, 85100 Potenza, Italy; nataschapascale@gmail.com (N.P.); aldodifaziomedicolegale@gmail.com (A.D.F.); 3 Azienda Ospedaliera Regionale “San Carlo”, Via Potito Petrone, 85100 Potenza, Italy; angela.bellettieri@ospedalesancarlo.it

**Keywords:** risk management, safety walkarounds, patient engagement

## Abstract

*Background and Objectives*: Healthcare facilities are complex systems due to the interaction between different factors (human, environmental, management, and technological). As complexity increases, it is known that the possibility of error increases; therefore, it becomes essential to be able to analyze the processes that occur within these contexts to prevent their occurrence, which is the task of risk management. For this purpose, in this feasibility study, we chose to evaluate the application of a new safety walkaround (SWA) model. *Materials and Methods*: A multidisciplinary working group made up of experts was established and then the subsequent phases of the activity were divided into three stages, namely the initial meeting, the operational phase, and the final meeting, to investigate knowledge regarding patient safety before and subsequently through visits to the department: the correct compilation of the medical record, adherence to evidence-based medicine (EBM) practices, the overall health and the degree of burnout of the various healthcare professionals, as well as the perception of empathy of staff by patients. *Results*: This working group chose to start this pilot project in the vascular surgery ward, demonstrating the ability of the tool used to capture the different aspects it set out to collect. In detail, the new version of SWA proposed in this work has made it possible to identify risk situations and system vulnerabilities that have allowed the introduction of corrective tools; detect adherence to existing company procedures, reschedule training on these specific topics after reviewing, and possibly update the same procedures; record the patient experience about the doctor–patient relationship and communication to hypothesize thematic courses on the subject; evaluate workers’ perception of their health conditions about work, and above all reassure operators that their well-being is in the interest of the management of the healthcare company, which is maintained. *Conclusions:* Therefore, the outcome of the present study demonstrates the versatility and ever-present usefulness of the SWA tool.

## 1. Introduction

In the WHO 2021 report on the trend of global healthcare spending in the previous two decades, it was observed that it had more than doubled, reaching USD 8.5 trillion in 2019, or 9.8% of global GDP, although unequally distributed, with high-income countries accounting for about 80%. In the HIC29 group (29 countries classified as high-income: Australia, Austria, Belgium, Canada, Cyprus, Denmark, Finland, France, Germany, Greece, Ireland, Israel, Italy, Japan, Kuwait, Luxembourg, the Netherlands, New Zealand, Norway, Portugal, Qatar, Singapore, Slovenia, Spain, Sweden, Switzerland, the United Arab Emirates, the United Kingdom, and the United States of America), per capita healthcare spending averaged USD 4491 and accounted for 9% of GDP in 2019, compared to USD 2923 in real terms or 7% in 2000 [1]. Health spending in low-income countries was financed mainly by direct spending (OOPS; 44%) and external aid (29%), while public spending dominated in high-income countries (70%). In low- and middle-income countries, on average, two-thirds of external aid went to healthcare for infectious diseases.

Healthcare facilities notoriously represent complex systems in which multiple professional experiences are provided to patients, each with their own peculiarities, intersecting with increasingly diversified and specialized technologies, surgical techniques, and pharmacological developments in a context that requires characterized economic sustainability by high volumes of services, staff who are not always numerically adequate, and reduced hospital stays [2]. In this context, the risk of error is possible.

“An error is defined as the failure of a planned action to be completed as intended or the use of a wrong plan to achieve an aim” [3], and “an adverse event is an injury caused by medical management rather than the underlying condition of the patient. An adverse event attributable to error is a preventable adverse event” [4].

If an error and therefore its risk become part of a process, the process can consequently be analyzed, the risk identified, and the error prevented in the dimension of clinical governance and risk management. This need arises from the consideration that healthcare companies can no longer limit themselves to the provision of care but have an obligation to provide patients with safe care—that is to say care that does not cause harm to patients [5]. For this to happen, a cultural change is required within healthcare organizations, an organizational management rethinking, which moves towards a blame culture which has its roots in a hierarchical management system. This seeks the fault and responsibility of those who commit an error, essentially seen as damage to a just or learning culture, with the involvement of employees in decision making policies, as well as the recipients themselves, with an attitude that considers the error as the failure of a process that must be known, analyzed, and from which you can learn [6]. In a just culture system, healthcare workers share responsibility and communicate openly, are trained on clinical risk topics, plan moments of discussion, analyze existing clinical practices from the perspective of constant improvement, and share information and experiences. These actions strengthen the quality of care provided, encouraging and increasing the number and quality of the provided reports.

In this regard, it should be noted that Italy is one of the countries that has a law that establishes and regulates rights and duties in the field of risk management. Law 24/2017 (known as the Gelli-Bianco law) reports in article 1: “*Safety of healthcare. 1. Safety of care is a constitutive part of the right to health and is pursued in the interest of the individual and the community. 2. The safety of care is also achieved through the set of all activities aimed at preventing and managing the risk associated with the provision of healthcare services and the appropriate use of structural, technological, and organizational resources. 3. All personnel are required to participate in the risk prevention activities implemented by public and private health and social-health facilities …*” [7]. This regulatory provision, therefore, translates the safety of care as a responsibility to which every healthcare professional is subjected and places the patient at the center of a “harmless” healthcare system, focusing attention on the prevention of errors to entirely guarantee the right to health, already constitutionally safeguarded. Thanks to the Gelli-Bianco law, healthcare and socio-health structures have developed greater sensitivity to risk management issues. Healthcare workers and hospitals are considered organically, since the former work in an organizational, structural, and technological context that depends on the latter.

As a consequence, the implementation of risk management has undergone methodological systematization over the years so that tools for risk assessment and risk analysis can be found. Among the risk assessment tools is the safety walkaround (SWA). First introduced by Frankel et al. [8], this tool is based on visiting different wards of the hospital, asking specific questions about adverse events or near misses that have occurred and been intercepted, and about the factors or system elements that might have led to these events. Subsequently, we witnessed the introduction of standardized questionnaires whose normalized results could be compared between different departments or in the same department over time following the introduction of corrective elements [9]. Nowadays, there is also the possibility of studying, through SWA, the processes that work, so as to be able to increase the awareness of operators [10].

In this context, the purpose of the present study is to evaluate whether the SWA, implemented and adapted for the new needs of complex healthcare systems, can be a useful tool for identifying the risk of error in healthcare. A daily evaluation of healthcare personnel’s habits by a group of experts can identify biases in the operational process and introduce improvements that encourage adherence to good clinical practices. In addition, healthcare workers will be involved in the risk management process, which will promote a sense of belonging to the system, trust, and non-blame.

A narrative review of the clinical risk methodology is also proposed in order to better understand the overall framework within which this study can be framed.

## 2. Materials and Methods

A multidisciplinary working group made up of experts was established. This group comprised the following:

- A physician director from the healthcare (HC) management and the clinical risk manager of the hospital as group coordinators;
- A specialist in forensic medicine to evaluate issues relating to informed consent and the correct compilation of health documentation;
- An external expert with expertise in medical error research for operational support;
- Nursing staff for the assessment of nursing activities;
- A pharmacist for the examination of potentially dangerous practices inherent to the process of distribution, storage, and administration of drugs;
- The occupational doctor of the company for the prevention of workplace risks;
- A psychologist expert in burnout assessment for the analysis of behavioral and emotional variables;
- A patient representative proposed by patient associations, involved in measuring the perception of the patient population.

The working group, thus constituted, then planned the subsequent phases of the activity by breaking them down into three stages: the initial meeting, the operative phase, and the final meeting.

During the initial meeting, a questionnaire (Annex S1) was administered to evaluate knowledge and skills in the field of clinical risk or to solicit any already known issues to be explored further, and the activity that would be carried out was explained.

For the second operational stage, over the course of three weeks, the different elements of the working group went separately to identify the specific characteristics of their expertise. In detail, the specialist in forensic medicine analyzed medical records and informed consent via a structured form, and the clinical risk manager analyzed adherence to EBM practices (including hand washing and prevention of falls in hospital settings). Using semi-structured interviews and questionnaires, the psychologist assessed the burnout of various healthcare professionals (doctors, nurses). The occupational doctor evaluated any risks associated with the health of workers based on the typical activities of the department. The patient representative, meanwhile, administered the Consultation and Relational Empathy Scale (CARE) questionnaire to evaluate PREMs (Patient Reported Experience Measures) [11,12,13], chosen for its simplicity of application based on 10 items and approved for the evaluation of empathy and the patient-centered approach.

Finally, in the final stage, in addition to an exchange of feedback on any errors or virtuous aspects detected, through a questionnaire (Annex S2) the perception of the usefulness of the experience and any aspects to be explored further following the experience were revealed.

This project involved two departments: vascular surgery and internal medicine. Study participants in the HCW group included all medical and nursing staff, and medical and nursing services were analyzed. All other healthcare professions (physiotherapists and technicians) were excluded. The sample of patients included subjects aged between 18 and 65 years in order to limit cultural biases coming from ages exceeding the selected range, with a hospital stay of ≥5 days so as to be able to express an informed evaluation of the hospital experience. At the end of the selection process, 50 HCWs and 50 patients were enrolled.

## 3. Results

This working group chose to start this pilot project in the vascular surgery ward (as a surgical example) and internal medicine ward (as a clinical model) as they have a smaller number of beds and therefore fewer healthcare workers and patients, with a turnover such as to be able to allow re-observation of some cases, as well as the study of multiple cases over three weeks. The preliminary meeting revealed that the culture of patient safety and risk management is still not widespread, and above all, the climate was one of mistrust and suspicion (Table S1). Indeed, to the question, “How do you consider your preparation/competence on the topic of clinical risk?”, 98% of the HCWs involved responded with “Poor” or “Sufficient”. The obstacle to undertaking the project (question 8) was identified as “difficulty in comparison” in 68% of cases, while the remainder attributed the difficulty to “mistrust, fears of possible return actions”.

During the subsequent operational phase, the following results were obtained:

- The analysis of medical records showed incomplete compilation of the medical records;
- The study of forms related to informed consent within the medical records indicated that insufficient information given to patients and insufficient completion of the forms were present;
- The evaluation of the application of the procedures relating to the 19 recommendations of the Italian Ministry of Health showed sufficient compliance with EBM practices;
- The specialist psychological evaluation concluded a not critical level of burnout despite operating volumes;
- The CARE questionnaire revealed that all (100% of 50 patients) of the interviewees measured a level of satisfaction no lower than “Very Good” and “Excellent” in both departments, confirming HCW dedication for willingness to dialogue with patients and optimal level of empathy in the relationship with the patient.

During the final meeting, these results were presented. Furthermore, new consent forms, prepared by the working group based on the guidelines of the relevant scientific societies, were presented for review by specialists in the sector, as well as training in the field of quality of health documentation. On the other hand, the staff, in a more relaxed and collaborative climate, provided their experience by proposing training topics in the clinical risk field and the usefulness of discussion also with the psychologist. This perception was quantitatively translated from the final questionnaire (Table S2), where to the question, “How much did you like the activity carried out?”, all the staff involved gave the answer “Good” or “High”. Furthermore, to the questions, “Are you available to repeat this type of experience in the future?” and “Do you think it could be useful to carry out this type of activity also for HCWs from other departments?”, all the answers were “Yes”.

## 4. Discussion

**Figure 1:**
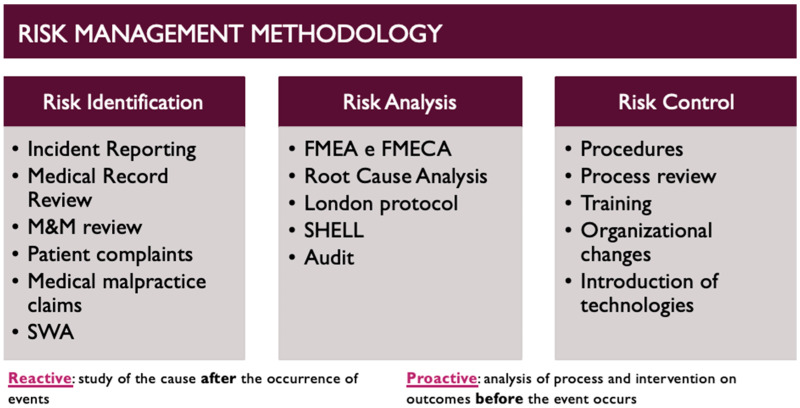
Risk management methodology.

Since risk management was introduced in the healthcare setting, it has developed over time into a working methodology that passes through three stages: risk identification, risk analysis, and risk control (Figure 1).

Incident reporting, medical record review, morbidity and mortality review, patient complaints and medical malpractice claims management, and SWA all represent risk assessment tools [14], while root cause analysis (RCA), London protocol, audit, failure mode and effect analysis/failure mode, effects, and criticality Analysis (FMEA/FMECA) [15], and SHELL are analysis methods.

Incident reporting involves the reporting of a possible risk, a near miss, or “an error that has the potential to cause an adverse event (patient harm) but fails to do so because of chance or because it is intercepted” [16], carried out by healthcare professionals. This information which collects clinical data and hypotheses of favorable factors can also be drawn up anonymously, demonstrating the promotion of a non-blame culture but also an interest in having the possibility of studying the process to support operators. The limits of this tool are represented by the following: (1) although it is widespread in many systems [17], in others where the culture of responsibility and guilt is historically more intrusive, reports are still very few; (2) some authors believe it has a low sensitivity in identifying patient safety incidents, particularly those resulting in harm [18].

A morbidity and mortality review, historically introduced in surgical settings [19], is a peer review of medical records of adverse events such as death or complications that happened to a patient during hospitalization. In detail, it consists in assessing the management and comparing it to best evidence or guidelines from the literature to evaluate its appropriateness or to identify potential failures that may have contributed to the development of the complication [20,21,22].

As far as medical record review is concerned, a premise must be made. According to the definition given by the Italian Ministry of Health, it is “the information tool designed to record all relevant demographic and clinical information on a patient during a single hospitalization episode”; therefore, its careful study provides information both from the chronological reconstruction of the clinical event and with respect to the quality of its compilation [23]. Research conducted by Brenner et al. demonstrated that medical record review had a sensitivity of 80% for discovering adverse events and a sensitivity of 76% for discovering negligent care [24]. The review of medical records allows to identify, within the documentation of an operational unit or patients involved in an error, a trigger process, which will then be analyzed by a team of experts in discriminating between complication and error. Medical records also provide various administrative information including hospital discharge forms. On SDO, the Agency for Healthcare Research and Quality (AHRQ) has identified PSI indicators (patient safety indicators) which, based on the coding of primary and secondary diagnoses according to International Classification of Diseases (ICD) codes, allow the assessment of a cohort being studied (i.e., post-surgical infections, pressure ulcer rate) [25,26]. The limit is represented by inadequate or inappropriate coding which excludes some cases from the analysis.

Patient complaints and medical malpractice claims management [27] can be a further source of potential information on the safety of care provided. The quantity and the resulting analysis of medical records in relation to the scientific evidence existing at the time of the facts, depositions, expert reviews, and adjustor notes represent, in fact, further ideas for evaluation [28].

Finally, the SWA represents a well-known and fundamental risk assessment tool. The purpose of this tool is to collect as much information as possible on risks, errors, near misses, or malfunctions of the system so as to be able to generate a system that learns and evolves from itself through process analysis and the production of corrective measures or awareness and promotion of virtuous behaviors.

In the context thus outlined, the new version of SWA proposed in this work has made it possible to identify risk situations and system vulnerabilities, which has allowed the introduction of corrective tools (information and informed consent compliant with the guidelines of specific national and international scientific societies) as well as the designing of a training program for all healthcare workers. It has also allowed us to detect adherence to existing company procedures and, if lacking, reschedule training on these specific topics after reviewing and possibly updating the same procedures, as well as promote effective processes by clearly presenting them to the various healthcare professionals so that they become aware of them and initiate benchmarking policies with departments lacking in these aspects. Further, this approach allowed us to record the patient experience in order to define the management characteristics of the doctor–patient relationship and communication within that department to hypothesize thematic courses on the subject, collect the perception of the activity carried out by the operators in terms of work-related stress and burnout by introducing a permanent clinic within the hospital for discussion between healthcare workers and a psychologist if they recognize the need, and reassure operators that their well-being is in the interest of the management of the healthcare company, which is maintained.

Knowledge of these tools is essential to avoid falling into the suggestion already summarized by the Joint Commission’s adage, “What You See Depends on How You Look” [29]. In fact, the use of a single tool during the risk assessment phase is not ideal as it would have the great limitation of collecting only a small percentage of serious incidents. Above all, there would be the risk of missing minor events or “near events”, and the frequency of data generated cannot track changes in safety over time, also because new introductions could modify the causal sequence of events, leading to a change in risks that may not be intercepted. Therefore, only the integration of all the tools will be able to guarantee a consistent view of the risks and events of the entire healthcare organization [30].

In our study, particular attention is paid to the experience of patients and human factors, from a person at the center of the work system perspective, be it healthcare workers and patients or an individual, with characteristics that include physical characteristics (strength of healthcare personnel, pathology of the patient), cognitive characteristics (expertise and experience of healthcare personnel, knowledge of the patient), and psychosocial characteristics (motivation of the healthcare professional, need for social support of the patient) [31]. The particular attention paid to patient involvement was also inspired by the Global Patient Safety Action Plan 2021–2030, “Towards eliminating avoidable harm in health care”, which is a document that provides a framework for different nations to develop their respective national action plans on patient safety. Created by the WHO with Member States and all relevant stakeholders, it has the desirable aim of eliminating avoidable harm in healthcare with the vision of “*a world in which no one is harmed in health care, and every patient receives safe and respectful care, every time, everywhere*” [32]. This plan is structured through various strategic objectives: number 4 is entitled “Patient and family engagement”, which has the aim of recalling the involvement and empowerment of patients as the most decisive tool for improving patient safety, who not only represent the users, no longer the object to which the care is directed, but subjects acting in cooperation with healthcare professionals, whose experiences represent a powerful tool for learning about the quality of the services provided. It is precisely this aspect that represented the real innovation of the present study compared to previous ones, with the result of increasing operator awareness of “knowing how to act”.

Once the risks have been identified, the subsequent analysis will be able to articulate a second system need. RCA is a reactive systematic analysis method following an adverse event, aimed at tracing the event back to its root causes, carried out by a multidisciplinary group [33]. The methods used are the Ishikawa or fishbone model, the Five Why scheme, and the process map. The limit is represented by the enormous deployment of resources in terms of time and human resources; therefore, it should be reserved for particularly serious events such as sentinel events [34].

The London protocol, like the RCA, is a systematic reactive technique; however, unlike the latter, it does not trace the event back to a few root causes but organizes the analysis according to patient characteristics, factors related to the operator, factors related to the team, factors related to the environment, and factors linked to the institutional context [35].

Proactive analysis tools are represented by qualitative FMEA and quantitative FMECA. They consist of the work of a multidisciplinary group, which, after examining the literature, health documentation, and expert opinion, breaks down a process into macro-activities. Each macro-activity is in turn divided into multiple tasks involved, and for each task, an error/failure mode is identified (FMEA) [36]. For each error, the FMECA adds an RPN (risk priority number) numerical value which identifies the analysis and intervention priorities [37]. Usually used for new processes to be implemented, it has a limitation represented by the fact that it analyzes phenomena individually, while it is well known that in complex systems, it is often the interaction between multiple aspects that represents the cause of an adverse event.

The SHELL model, introduced in the aviation sector and then extended to the healthcare sector like many elements of the clinical risk methodology, places the human factor at the center of the analysis as a basic component of a socio-work institutional fabric, and looks at the mutual interactions with (i) the rules, standards, and training that regulate conduct (Software—S); (ii) the tools and technologies that are used by man during routine work (Hardware—H); (iii) the physical and social working context (Environment—E); and (iv) the behavior of other human beings who work and operate at different organizational levels (Liveware—L). The centrality of the human factor is therefore confirmed by the direction of the analysis, aimed at questioning the relationships among healthcare professionals (medical and non-medical) and know-how deriving from specialist training as well as from legal and disciplinary rules (L-S); healthcare professionals and tools used to carry out their activity, including drugs, surgical instruments, machinery, and ergonomics (L-H); healthcare professionals and overall hospital context, also understood as socio-working climate (L-E); and interaction and cooperation with other colleagues or professionals of another type or branch (L-L). Once the most deficient relationship has been identified, it will guide the introduction of corrective measures [38,39].

As far as audit is concerned, it is appropriate to distinguish between a clinical audit (proactive, quantitative) [40] and an SEA (reactive, qualitative) [41]. The latter represents a meeting of professionals who in various capacities have followed one another in a case that was the subject of an adverse event (significant event audit), and they ask themselves what went wrong in the case in question. The outcome of the analysis can lead to preventive measures aimed at preventing the event from recurring. A clinical audit, on the other hand, originates from the choice of a topic of particular interest for an operational unit (because it is linked to high volumes or high mortality and morbidity). After this, a multi-disciplinary group is formed which, through a review of the literature, will identify performance indicators (outcome or process) which will then be measured in the normal clinical practice of the same operational unit to evaluate deviation. In the presence of any deviation, corrective measures will be introduced, the effectiveness of which will be assessed by re-measurement of the same indicators. It is a procedure that respects the quality cycle, beginning with an audit and ending with a re-audit.

Furthermore, a recent review of the literature has highlighted the possibility of applying artificial intelligence (AI) as a support to the clinical risk methodology in order to facilitate the identification of adverse events, errors, near misses, and risks [42]. In particular, this study underlined the possibility, through codified algorithms, of identifying cases at risk of developing surgical site infections or sepsis in an early manner which could go undetected by healthcare workers. The objective would be to nominate them and promptly initiate them into the Hour-1 Bundle, thus having a considerable impact on the reduction in morbidity- and mortality-related infections [43]. Another sphere of interest is incident reporting. One of the main limitations of the effectiveness of the incident reporting system lies in the lack of use of codified terminology in the reporting of adverse events by healthcare professionals, which leads to a lack of aggregation of data with consequent underestimation of adverse events. AI has proven useful in increasing the efficiency of this risk identification tool by converting free text into structured information, as well as in reducing workload for healthcare professionals.

Despite all the techniques presented, and also in light of the limitations highlighted, in recent years it has been hypothesized that the main obstacle, in addition to the recentness of the discipline of patient safety, consists precisely in the methodology, and some authors support the need to move from “Patient Safety I” (PS-I) to “Patient Safety II” (PS-II) [44]. In the editorial entitled “*False Dawns and New Horizons in Patient Safety Research and Practice*”, Mannion and Braithwaite, underlining the excessive optimism with which the introduction of this subject had been welcomed and the naivety of the first approaches, call for a radical change in the system of thought [45]. No longer just and simply just culture, they go much further and further. Framing safety as the absence of damage, according to the dictates of the PS-I, maintains the negative connotation around human error or system failure. Alternatively, the PS-II is revolutionary in its affirmative and statistically more relevant prerogative with reference to the ability to obtain positive results in the majority of cases, despite the stresses and tension present in any complex system, such as the healthcare one. Specific strategies or approaches of “Patient Safety II” should investigate daily work starting from traditional safety issues, among which some authors identify the management of preoperative drugs or intravenous infusions [46] and the work routine—that is, the question becomes, “what was so ordinary about this case”, instead of, “what was so extraordinary,” focusing on what this tells us about the work being conducted. Using this different approach shifts the focus from what went wrong and what may have failed towards the trade-offs and adaptations people need to make and how these could be supported. The different approach inevitably involves greater attention and valorization of the human factor.

Despite being a recent subject, this risk management methodology has been defined as traditional, in contrast, and can be compared with new and more modern systems, including enterprise risk management (ERM) [47]. ERM is a strategic approach to improve the quality of service, which in hospitals translates to improved performance, through the analysis of risks and economic advantages. In other words, hospital management must take into account the complexity of the financial structure of a hospital, consisting of direct and indirect costs and sources of revenue; improve the financial health; and implement hospital safety management while minimizing costs and resources. It is a holistic model that aims to evaluate the overall level of risk and highlight any interactions present between the different types of risk, producing information for multiple recipients [48]. The Committee of Sponsoring Organization of the Treadway Commission (COSO) has defined “Enterprise Risk Management as a process, affected by an entity’s board of directors, management and other personnel, applied in strategy-setting and across the enterprise, designed to identify potential events that may affect the entity, and manage risk to be within its risk appetite, to provide reasonable assurance regarding the achievement of entity objectives” [49]. ERM introduces “risk appetite”, i.e., the level of acceptable risk for the pursuit of the objectives, defined by the analysis of the benefits deriving from the achievement of the objectives and the costs linked to the risks connected to them, through policies, methodologies, and infrastructure [47]. Policies should be consistent with the business strategy and communicated internally and externally to the organization. The methodologies represent the statistical–mathematical support that must be connected to performance management. Infrastructure represents the system of control that begins with training staff and ends with the production of risk reports.

Another methodology is lean management (LM), which starts from the assumption that organizations, including healthcare ones, are substantiated through processes—connected activities that have a specific order and space, with a clearly defined beginning and end, which can cross and interconnect with other subprocesses or form the start/end of another procedure [50,51]. In this context, lean means supporting “value” at every stage, where value is what a customer would pay for and waste is what a customer would not pay for; consequently, activities without added value (definable precisely in terms of waste), which often add delays, require additional resources, and attract additional costs, should be eliminated [52]. The transfer of lean management to the healthcare sector is relatively new [53,54]. The methodology is structured around five pillars: (a) specify the value from the patient’s point of view from admission to hospital until discharge; (b) identify the value stream for each product/service provided and counteract all wasted steps by mapping all processes involved in creating a product/service; (c) make the flow of products/services continuous and standardize processes around best practices; (d) introduce “traction” between all the phases in which continuous flow is impossible and aim to eliminate waste as much as possible by “dragging” the customer/patient, i.e., keeping in mind the next phase of the process; (e) manage towards perfection. For example, McCulloch et al. [55] applied the lean process to improve the safety care process in a surgical emergency unit, underlining that this methodology can lead to substantial improvements in the reliability of safety-critical care processes. Furthermore, lean methods can improve the diagnostic accuracy of some laboratory tests [56] and reduce the frequency of process-dependent errors [57].

Knowledge of the different techniques and tools [42] remains fundamental for a forensic doctor who wants to abandon his usual forensic field and apply the typical analytical methodology to the topic of risk in healthcare, operating within structures with their own uniqueness and complexity, which requires adaptation of different analysis models, as is the example of the SWA used for the present study.

## 5. Conclusions

Although this study has a limitation in its application within a single ward for a branch (clinic and surgical), used as a sample, the revision proposed in this pilot study of the SWA has demonstrated the usefulness of the tool that is able to adapt to the needs of new healthcare organizations to identify risks and aspects that can be improved but also positive processes, with greater awareness on the part of operators and greater compliance on the part of patients. In this constantly evolving context, despite the recentness of the subject, made up of methodologies being compared and promoted, the outcome of the present study demonstrates the versatility of the SWA tool. In addition to its well-known reactive attitude to analyzing problems, this tool has also demonstrated not only proactivity when conducted by a multidisciplinary group aimed at identifying risks within a process that has already demonstrated failure (sentinel events or adverse events in general) but also the possibility of intercepting risks during normal department activity, also including aspects such as burnout and the perception of patients’ experience. As previously mentioned, clinical risk management tools are different and applicable for different purposes, although each has its own limitations. They rely on human input, which can be subjective and prone to errors. For example, incident reporting systems may not capture all incidents, and the quality and accuracy of the data entered can vary depending on the person reporting. Similarly, root cause analysis and failure mode and effects analysis require a skilled and experienced team to conduct the analysis and make recommendations for improvement. Another limitation is that some tools may not be suitable for all types of healthcare settings or may require significant resources to implement and maintain. For example, some tools may be more appropriate for hospitals than for outpatient clinics or long-term care facilities. Additionally, some tools may require specialized training or software, which can be costly and time-consuming to implement. These data highlight the need for the implementation of integrated models in a context of constant updating and adaptation of the single tool to the individual healthcare reality.

## Data Availability

The original contributions presented in the study are included in the article/supplementary material, and further inquiries can be directed to the corresponding author(s).

## Supplementary Materials

The following supporting information can be downloaded at: https://www.mdpi.com/article/10.3390/medicina60060903/s1. Annex S1. Initial meeting questionnaire; Annex S2. Final meeting questionnaire; Table S1. Answers to Annex S1; Table S2. Answers to Annex S2.
