# Supplementary material for: Is It Still Time for Safety Walkaround? Pilot Project Proposing a New Model and a Review of the Methodology
Source: Medicina (Kaunas). 2024 May 29;60(6):903. doi: 10.3390/medicina60060903 (PMC11205543; doi:10.3390/medicina60060903)
Supplement: Supplementary file 1 [file medicina-60-00903-s001.zip › medicina-2985576-supplementary.pdf]

Annex S1. Initial meeting questionnaire

Qualification: Doctor Nurse

1) Have you already attended training courses on the topic of clinical risk?
Yes No

2) Are you familiar with this risk detection technique called Safety Walk Around?
Yes No

3) How do you consider your preparation/expertise on the topic of clinical risk?
Poor Sufficient Good High

4) How do you define your personal level of interest in the topic of clinical risk?
Poor Moderate Good High

5) How relevant do you think these issues are on a theoretical/speculative level?
A little Fairly A lot

6) How relevant do you think these issues are on a practical/operational level?
A little Fairly A lot

7) Do you think your work environment is open and sensitive to these issues?
A little Fairly A lot

8) Do you think there will be obstacles in your ward to undertake/develop this initiative to understand the problems related to risk perception and patient safety?
Yes No
If "yes", which one?
Distrust, fears of possible reactions Difficulty in confrontation

9) Do you think there will be improvements/corrections in the work/organization of your ward after this initiative?
Yes No

10) Do you believe that there will be worsening/negative measures in your ward after this initiative?
Yes No

11) Which of the following clinical risk management tools do you know and would be able to apply from today?
Audit Incident reporting FMEA Safety walk around
Root causes analysis

Thanks for collaboration.

Annex S2. Final meeting questionnaire

Qualification: Doctor Nurse

1) What was your satisfaction with the activity carried out?
Poor Sufficient Good High

2) Are you available to repeat this type of experience in the future?
Yes No

3) Do you think it could be useful to carry out this type of activity also for HCW in other wards?

Yes No

4) Do you think that further training on the covered topics is necessary?
Yes No
If "yes", which one?
General aspects of clinical risk management
Clinical risk management tools
Patient involvement during treatment - informed consent
Health records

5) In light of your experience, how relevant do you think these issues are on a theoretical/speculative level?
A little Fairly A lot

6) In light of your experience, how relevant do you think these issues are on a practical/operational level?
A little Fairly A lot

7) Have you received information on the outcome of the SWA activity carried out?
Yes No

8) Do you believe that the results of the activity carried out will be taken into consideration by the managers/directors of your ward/HC facility?
Yes No

9) To your knowledge, have any improvements/corrections been introduced in the work and/or organization of your department after this initiative?
Yes No
If "yes", which one?

Thanks for collaboration.

Table S1. Answer to Annex S1

Doctor (D) 30% 15	Nurse (N) 70% 35

Item 1	Yes<br>D 4%<br>N 4%	No<br>D 26%<br>N 66%

Item 2	Yes<br>D 4%<br>N 4%	No<br>D 26%<br>N 66%

Item 3	Poor<br>D 8%<br>N 30%	Sufficient<br>D 20%<br>N 40%	Good<br>D 2%<br>N 0	High<br>D 0<br>N 0

Item 4	Poor<br>D 0<br>N 0	Moderate<br>D 4%<br>N 10%	Good<br>D 26%<br>N 60%	High<br>D 0<br>N 0

Item 5	A little<br>D 12%<br>N 40%	Fairly<br>D 14%<br>N 30%	A lot<br>D 0<br>N 0

Item 6	A little<br>D 14%<br>N 50%	Fairly<br>D 12%<br>N 20%	A lot<br>D 0<br>N 0

Item 7	A little<br>D 20%<br>N 54%	Fairly<br>D 10%<br>N 16%	A lot<br>D 0<br>N 0

Item 8	Yes<br>D 14%<br>N 62%	No<br>D 16%<br>N 8%	Difficulty 68%<br>D 10%<br>N 58%	Distrust 8%<br>D 4%<br>N 4%

Item 9	Yes<br>D 26%<br>N 54%	No<br>D 4%<br>N 16%

Item 10	Yes<br>D 0<br>N 10%	No<br>D 30%<br>N 70%

Item 11	Audit<br>D 12%<br>N 20%	Incident<br>D 0<br>N 10%	FMEA<br>D 0<br>N 0	SWA<br>D 0<br>N 0	RCA<br>D 0<br>N 0

Table S2. Answer to Annex S2

Doctor (D) 30% 15	Nurse (N) 70% 35

Item 1	Poor<br>D 0<br>N 0	Sufficient<br>D 0<br>N 0	Good<br>D 14%<br>N 18%	High<br>D 16%<br>N 52%

Item 2	Yes<br>D 30%<br>N 70%	No<br>D 0<br>N 0

Item 3	Yes<br>D 30%<br>N 70%	No<br>D 0<br>N 0

Item 4	Yes<br>D 30%<br>N 70%	No<br>D 0<br>N 0	General<br>D 2%<br>N 16%	Clinical<br>D 2%<br>N 12%	Patient<br>D 18%<br>N 6%	Records<br>D 8%<br>N 36%

Item 5	A little<br>D 4%<br>N 8%	Fairly<br>D 6%<br>N 26%	A lot<br>D 20%<br>N 36%

Item 6	A little<br>D 4%<br>N 8%	Fairly<br>D 6%<br>N 20%	A lot<br>D 20%<br>N 42%

Item 9	Yes<br>D 24%<br>N 54%	No<br>D 6%<br>N 16%

Item 10	Yes<br>D 0<br>N 10%	No<br>D 30%<br>N 70%
